# The 3 R’s for Platelet-Rich Fibrin: A “Super” Tri-Dimensional Biomaterial for Contemporary Naturally-Guided Oro-Maxillo-Facial Soft and Hard Tissue Repair, Reconstruction and Regeneration

**DOI:** 10.3390/ma11081293

**Published:** 2018-07-26

**Authors:** Consuelo C. Zumarán, Marcelo V. Parra, Sergio A. Olate, Eduardo G. Fernández, Francisco T. Muñoz, Ziyad S. Haidar

**Affiliations:** 1 BioMAT’X, Facultad de Odontología, Universidad de los Andes, Santiago 7550000, Chile; czumaran@uandes.cl (C.C.Z.); fmunoz.thomson@gmail.com (F.T.M.); 2 CEMyQ, Facultad de Odontología, Universidad de La Frontera, Temuco 4780000, Chile; marcelo.parra@ufrontera.cl (M.V.P.); sergio.olate@ufrontera.cl (S.A.O.); 3 Departamento de Odontología Restauradora, Facultad de Odontología, Universidad de Chile, Santiago 8320000, Chile; edofdez@yahoo.com; 4 Instituto de Ciencias Biomédicas, Universidad Autónoma de Chile, Santiago 7500912, Chile; 5 Programa de Especialización en Cirugía Bucal y Maxilofacial, Facultad de Odontología, Universidad de los Andes, Santiago 7550000, Chile; 6 Programa de Doctorado (BioMedicina), Facultad de Medicina, Universidad de los Andes, Santiago 7550000, Chile; 7 Centro de Investigación e Innovación Biomédica (CIIB), Facultad de Medicina, Universidad de los Andes, Santiago 7550000, Chile

**Keywords:** tissue engineering, regeneration, leukocyte, platelet, fibrin, growth factors, dentistry, oral surgery, periodontology, osteogenesis, grafts

## Abstract

Platelet-Rich fibrin (PRF) is a three-dimensional (3-D) autogenous biomaterial obtained via simple and rapid centrifugation from the patient’s whole blood samples, without including anti-coagulants, bovine thrombin, additives, or any gelifying agents. At the moment, it is safe to say that in oral and maxillofacial surgery, PRFs (particularly, the pure platelet-rich fibrin or P-PRF and leukocyte and platelet-rich fibrin or L-PRF sub-families) are receiving the most attention, essentially because of their simplicity, cost-effectiveness, and user-friendliness/malleability; they are a *fairly* new “revolutionary” step in second-generation therapies based on platelet concentration, indeed. Yet, the clinical effectiveness of such surgical adjuvants or regenerative platelet concentrate-based preparations continues to be highly debatable, primarily as a result of preparation protocol variability, limited evidence-based clinical literature, and/or poor understanding of bio-components and clinico-mechanical properties. To provide a practical update on the application of PRFs during oral surgery procedures, this critical review focuses on evidence obtained from human randomized and controlled clinical trials only. The aim is to serve the reader with current information on the clinical potential, limitations, challenges, and prospects of PRFs. Accordingly, reports often associate autologous PRFs with early bone formation and maturation; accelerated soft-tissue healing; and reduced post-surgical edema, pain, and discomfort. An advanced and original tool in regenerative dentistry, PRFs present a strong alternative and presumably cost-effective biomaterial for oro-maxillo-facial tissue (soft and hard) repair and regeneration. Yet, preparation protocols continue to be a source of confusion, thereby requiring revision and standardization. Moreover, to increase the validity, comprehension, and therapeutic potential of the reported findings or observations, a decent analysis of the mechanico-rheological properties, bio-components, and their bioactive function is eagerly needed and awaited; afterwards, the field can progress toward a brand-new era of “super” oro-dental biomaterials and bioscaffolds for use in oral and maxillofacial tissue repair and regeneration, and beyond.

## 1. Introduction

Even though significant improvements in restoration and/or replacement approaches and supplies have been accomplished over the last decades, the repair and regeneration of defects remains a challenge [1]. Indeed, current clinical approaches that have been used to reconstruct and heal *complex* defects, including different *multi-disciplinary* methods of bone grafting, such as autologous bone grafts, distraction osteogenesis, allografts, bone-graft substitutes, and/or guided bone regeneration, are deemed restricted on a daily basis. This is often multi-factorial; whether due to the limited self-renewal capacity of the defect and/or the limited donor supply, increased morbidity, risk of antigenicity, and foreign body reactions associated with the grafts used. While several oro-dental soft and hard tissues have been regenerated using mesenchymal stem cells (oro-dental sources mainly), the translation of novel biomaterials to the clinic has been slow. This is attributed to a lack of scientific knowledge and ethical considerations. Overall, operative-associated time and cost contribute as well. Hence, the field of dentistry (in general, and oral surgery specialties, in particular) is anticipated to significantly transform in the next two to three decades via the introduction of innovative tissue engineering and/or tissue-engineered bio-products. This boom will include various oro-dental soft and hard tissues including enamel, dentin, alveolar jaw bone, periodontium, oral and buccal mucosa, as well as the salivary glands. Thereby, the art and science of oro-maxillo-facial reconstruction is of great interest for contemporary oral and maxillofacial surgeons as part of the search for better bioengineering strategies and biomaterials, which is a core driver for bio-dental research today [2]. Platelet concentrates are a fine example.

**Figure 1:**
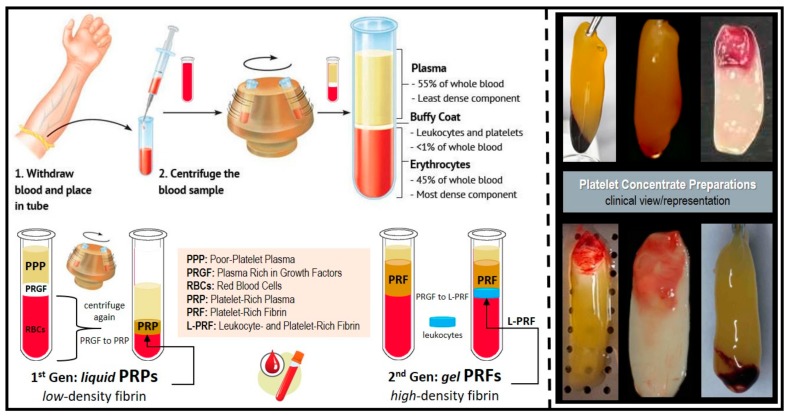
Platelet concentrates’ clinical preparation, types/classes, and clinical illustration/presentation of several platelet-rich fibrin (PRF) and leukocyte and platelet-rich fibrin (L-PRF) preparations (membranes).

In simple words, the ‘product’ resulting from the centrifugation of whole blood samples are autologous blood extracts, so-called *platelet concentrates* (Figure 1). In clinically-usable preparations (surgical adjuvants), the preparation procedure may enhance, accelerate, and promote tissue (soft and hard) wound healing and regeneration due to its potential to allow the gathering and concentration of platelets and other therapeutic blood constituents (fibrinogen/fibrin, growth factors, leukocytes, and circulating cells) [3]. Currently, their overall effectiveness remains debated, despite promising clinical observations. The main reasons are: contradictory clinical outcomes, insufficient high-quality evidence-based literature, and poor identification of the characteristics of the end-products (and preparation protocols) used in research studies; along with—until recently—a lack of proper nomenclature to typify these concentrates [4]. In fact, in 2009, the first “classification” consensus [5] was published, categorizing four particular platelet concentrate sub-families relying on differences in biological components (fibrin and cell), properties (gelification), and possible applications: pure platelet-rich plasma (P-PRP), leukocyte and platelet-rich plasma (L-PRP), pure platelet-rich fibrin (P-PRF), and leukocyte and platelet-rich fibrin (L-PRF) [5]. Nowadays, when compared to the PRPs, it can be stated that in oral and maxillofacial surgery, the PRFs (P-PRF and L-PRF sub-families; not including red blood cells within) [4,5,6] are receiving the highest consideration and hype, which is mainly due to their preparation simplicity and rapidness, user-friendliness/malleability, and *likely* cost-effectiveness.

**Figure 2:**
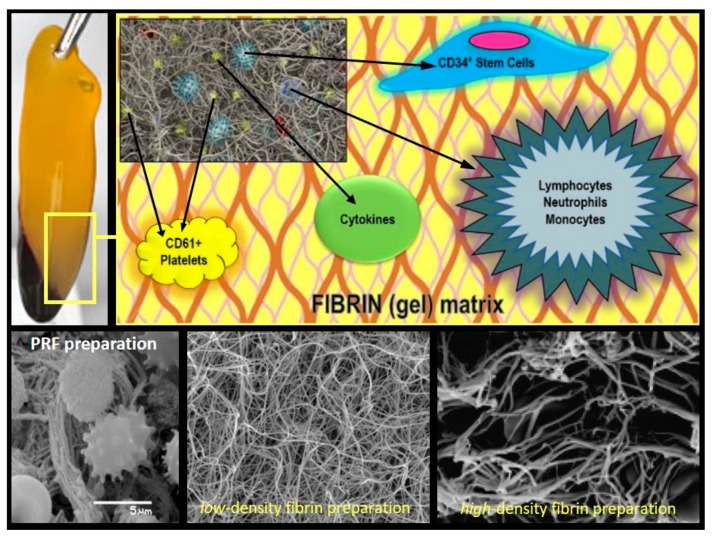
PRF Composition/Architecture Illustration. Schematic representation of PRF bio-components and SEM (scanning electron microscope) micrographs of the PRF membranes displaying its polymerized interconnected fibrin network and large living cell population content.

PRF and L-PRF are second-generation autologous platelet concentrates of whole venous blood [4,6]. A fibrin gel that is polymerized slowly and strongly (Figure 2) abundant in growth factors, platelets, leukocytes (almost half of the initial blood harvest), and lymphocytes is collected, following simple and rapid (~10 min) centrifugation (*please note that preparation protocols vary*) of blood, in vacutainer tubes, without anti-coagulant. The gathered clot (or biomaterial) is stable, resilient, strong, adhesive, and malleable. It can be cut or adapted into different anatomical defects and applications: bended with bone grafting material, applied as filling material in a direct way, or compacted into a strong fibrin membrane. Alongside this established clinical ease of use and handling, the biochemical composition of the PRF by-products provides it with attractive hemostatic, angiogenic, osteogenic, anti-inflammatory, anti-microbial, pain-inhibitory, and wound-healing characteristics [3,7,8]. This critical review aims to provide the clinical reader with an up-to-date evidence-based presentation on the evaluation of PRFs’ (mainly, P-PRF and L-PRF) use and application for oro-maxillo-facial tissue regeneration, from high-quality randomized and controlled clinical trials. Thus, in vitro, in vivo, and *case report* studies were intentionally excluded from analysis.

## 2. Materials and Methods

**Figure 3:**
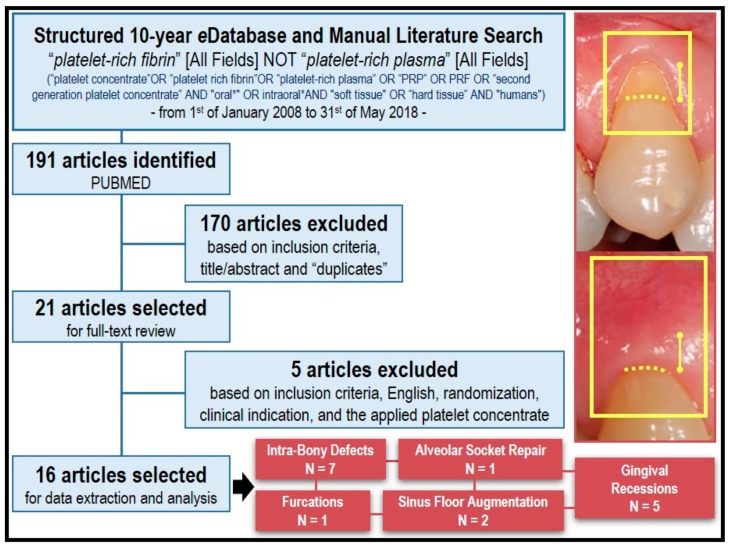
Flow chart of literature search strategy, hits, and included studies for data extraction and analysis. A clinical example illustrating the benefits of PRF application in treating gingival recession is displayed.

A systematic and structured literature search (Figure 3) conducted on PUBMED (January 2008–May 2018) including the MeSH (Medical Subject Headings) terms “platelet-rich fibrin” and “platelet-rich plasma” with the search strategy established as: “platelet-rich fibrin” [All Fields] NOT “platelet-rich plasma” [All Fields]. The filters that were used to limit the results were: Seniority (published 10 years ago: up to May 2018), Language (English), Availability (Full-text), and Species (Human). Inclusion criteria were: (a) randomized clinical trials (RCTs) using (b) Choukroun’s PRFs in (c) oro-maxillo-facial procedures. The initial search resulted in 191 articles, 21 of which met the inclusion criteria. Five articles were then excluded due to (exclusion criteria) quality, availability, and/or lack of randomization. Due to the high heterogeneity in the analyzed literature, the results are presented and discussed in a narrative format.

## 3. Results and Discussion

The initial search yielded 191 articles in PUBMED. After title, abstract, and text screening, 21 articles met the initial inclusion criteria. Google Scholar and hand searching the bibliography section of select reviews yielded similar results. After full-text analysis, five articles were excluded due to meeting the pre-established exclusion criteria, resulting in 16 studies (Table 1) that were opted for data extraction and analysis.

### 3.1. PRFs in the Periodontal Intra-Bony Defects (IBDs) Treatment

**Figure 4:**
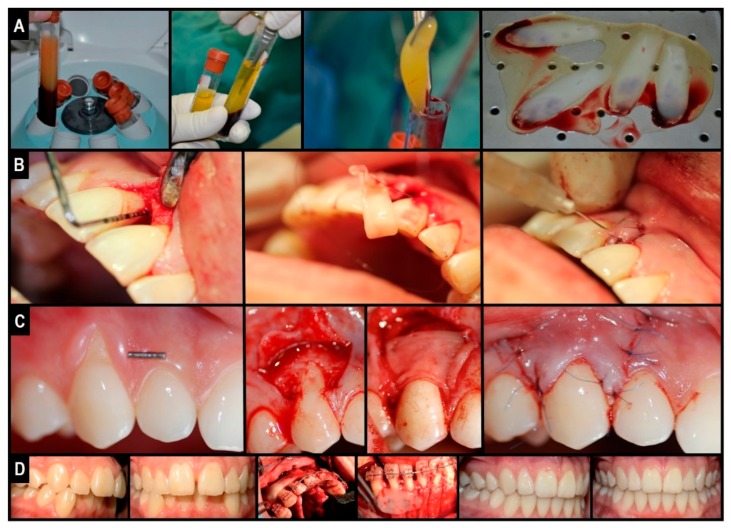
Clinical illustration of L-PRF application in oro-maxillo-facial surgery defect regeneration: natural guided tissue bio-engineering using L-PRF as a “bio-scaffold”. (**A**) L-PRF membrane preparation; (**B**) clinical application in IBDs or for the treatment of periodontal intra-bony defects; (**C**) Clinical application under CAF or coronally advanced flaps in the treatment of gingival tissue recession; (**D**) clinical application in PAOO, or periodontally accelerated osteogenic orthodontics—an orthognathic procedure.

The regeneration of periodontal tissue is conventionally known as the generation of new cementum, alveolar bone, and a functional periodontal ligament on a tooth-supporting root surface that is previously diseased. Due to limited intrinsic regenerative potential, intra-bony defects (IBDs) are a common and challenging sequel of periodontal disease. Meta-analyses demonstrated that treatment with conservative open flap debridement produces an average clinical attachment (CAL) gain of 2.0 mm [25]. While about 1.5 mm may be attributed to newly formed bone, bone-fill does not implicate the regeneration of new attachment to the root [25]. In this context, PRFs appear promising for the regeneration of the whole periodontal attachment system (Figure 4A,B). Seven RCTs addressing the prospective application of PRFs periodontal therapy IBD have been found. Articles that have been found allowed for the following comparisons: (a) PRF/open flap surgery versus open flap surgery [9,10,11]; (b) PRF/Bio-Oss^®^ constructs (Bio-Oss^®^, Geistlich Pharma North America, Inc., Princeton, NJ, USA) versus PRF [12] and (c) PRF/DFDBA constructs versus DFDBA (demineralized freeze-dried bone allograft) [13] (d) PRF/inorganic bovine bone mineral (ABBM) versus ABBM alone [14] and (e) resorbable collagen membrane + PRF versus guidance tissue regeneration [15]. All of the patients who were included in those studies were periodontally stable and systemically healthy individuals who presented: a similar bilateral IBD of at least 5-mm probing depth located in vital asymptomatic teeth with no furcation involvement. Studies evaluating the addition of PRF to a conventional open flap procedure reported the biomaterial notably improving both the clinical and radiographic parameters of IBDs, after nine [9,11] and 12 months [10]. A significant improvement in probing depth (PD) decrement, CAL increase, post-treatment gingival margin stability [(GMS) *less post-treatment gingival recession*], bone defect fill, and percentage bone defect fill were noticed in all of the PRF-treated sites versus controls [9,10,11]. Interestingly, higher patient acceptance was also associated with the use of PRFs. This is most likely attributed to the accelerated wound healing and pain-inhibitory properties [9,10]. The presented PD reduction and CAL gain values were superior to previously-reported values in a meta-analysis performed for open flap surgery [25], suggesting the additional advantages of PRFs over the conventional approach. Treatment with PRF/particulate bone-graft substitutes (Bio-Oss^®^ [12] and DFDBA [13]) provided additional statistically significant advantages regarding PD reduction, CAL gain, and bone defect fill versus graft substitutes after six months. Nonetheless, the absence of a “simultaneously-run” PRF-alone control renders it difficult to distinguish between the effects of PRFs and other potential variables in the study. Thus, while promising, additional studies are deemed essential in order to appropriately determine (quantifiably) the effectiveness and advantages of PRFs application over particulate bone-grafts use.

### 3.2. PRFs in the Periodontal Furcation Defects (PFDs) Treatment

From periodontitis, many molars result with furcation involvement, promoting higher rates of periodontal breakdown and poorer prognosis than single-rooted teeth [26]. Contemporary treatment options often include the use of regenerative materials and bone grafts; however, the introduction of PRFs seems promising for better therapeutic outcomes. In our analyses, one RCT addressing the therapeutic use of PRFs in PFDs was found [16]. The study compared PRF/open flap versus open flap debridgement alone in the therapy of grade-II mandibular discrepancies. Included patients were periodontally stable and systemically healthy, with similar bi-lateral grade II buccal furcation defects (at least 5 mm of probing depth and ≥3 mm of horizontal probing depth), in vital asymptomatic mandibular first molars. 

PRF use significantly improved clinical and radiographic parameters of conventional open flap debridement. After nine months, the defect’s complete clinical closure was successful in 66.7% of L-PRF-treated locations. Severity within residual defects was reduced in five out of six sites (degree I), whereas one defect remained in degree II. Significantly greater PD reduction, radiographic vertical defect fill, and CAL gain was reported on experimental sites versus controls. PRF use was also associated with a greater post-treatment GMS [16].

### 3.3. Miller Class I and II Gingival Recession Treatment by PRFs

Gingival recession is characterized by the root surface exposure caused by the gingival margin’s apical migration. If left untreated, the condition may lead to other problems including: deficient esthetics, dentine hypersensitivity, and a higher risk of dental caries [27]. Available treatment options include the application of: (a) coronally-advanced flaps (CAF); (b) connective tissue grafts (CTG); and (c) sub-epithelial connective tissue grafts (SCTG). On their own, the aforementioned techniques have important limitations such as (a) unpredictable long-term root coverage (i.e., CAF decreases from 89% to 58.8% after six months); (b) limited gain of keratinized tissue width (KTW), which is important to prevent recurrence; and (c) adverse post-surgical effects such as pain/discomfort, swelling, flap necrosis, etc. [27]. In this review, in the context of dental care of gingival recessions, seven RCTs evaluating the appliance of PRFs were identified and included. The investigations allowed for the following comparisons: (a) PRF/CAF versus CAF [17,18]; (b) PRF/CAF versus EMD (enamel matrix derivate)/CAF [19]; (c) PRF/CAF versus CTG [21]; and (d) PRF/CAF versus SCTG [20]. Similar to previous RCTs, all of the patients included herein were periodontally stable and systemically healthy; they presented with: similar bi-lateral Miller Class I or II gingival recessions (≥2 mm depth) localized on vital teeth, without restorations. According to Padma et al., the addition of PRF to CAF improved both clinical outcomes and the post-treatment stability of CAF [17]. After six months, the authors reported (significantly) more recession depth (RD) reduction, CAL gain, and KTW increase in all of the PRF-treated sites versus controls. Interestingly, post-treatment GMS was also higher in the test group with 100% root coverage after six months versus 64.88% in controls [17]. However, in contrary with this RCT, Aroca et al. reported limited clinical benefits when using the PRF/CAF approach [18]. Herein, CAL gain and gingival tissue thickness (GTH) were the only benefiters of the combination; when comparing CAF-alone controls to the test group, significantly higher percentage root coverage, full root coverage, GMS, and recession width (RW) reduction were found in the first group [18]. Such “*contradictory*” results may be partially explained by deficient study design, which not only failed to adequately include blind examiners (leading to potential bias in favor of the “traditional” approach), but also included: multiple adjacent gingival recessions (with poorer prognosis than single/localized recessions); heavy smokers (in which healing response is usually altered); and the PRFs were stored in a 4 °C refrigerator until use (PRFs protocols often recommend immediate/fresh use). Indeed, emerging evidence states that growth factor release from PRFs initiates as early as 5 min from preparation (centrifugation step mainly). Hence, storage could have altered its properties and thereby diminished or deteriorated its clinical potential. When compared with other root coverage procedures (EMD/CAF, CTG, and SCTG), the PRF/CAF approach showed similar clinical outcomes regarding RD reduction, CAL gain, mean root coverage (%), and complete root coverage (%). The only exception was KTW increase, with both EMD/CAF and CTG controls showing higher KTW than PRF-treated groups [19,20,21]. Interestingly, all of the studies reported significantly faster healing and fewer complications (pain and discomfort) when PRF was used [19,20,21]. The findings are notable, especially when comparing with SCTG (the current “*gold standard*” technique for managing Miller Class I and II gingival recessions), indicating that PRF/CAF could be a safer and less invasive alternative to current grafting techniques, and a more cost-effective strategy or approach than EMD in the treatment of Miller Class I and II gingival recessions (Figure 4C).

### 3.4. PRFs in Sinus Floor Augmentation

The resorption of the upper jaw bone after tooth loss is a frequent problem faced in posterior maxillary implant placement due to the lack of required bone mass for anchorage. Common maxillary sinus augmentation techniques provide a solution via increasing the available bone height at the expense of sacrificing volume of the maxillary sinus [28]. Traditionally, autologous bone grafts and resorbable membranes are used to promote osteogenesis and avoid soft tissue in-growth into the surgical site. However, donor site morbidity and size restrictions, the latter resorption of the graft, and the high cost of membranes are the main disadvantages [29,30]. In this context, PRFs appear to provide a promising alternative overcoming such limitations. In this review, two RCTs evaluating the use of PRFs in lateral window sinus augmentation were found. Applications were performed either as: (a) grafting material (PRF/Bio-Oss^®^ constructs versus Bio-Oss^®^) [22] or (b) absorbable covering membrane for the lateral osteotomy window (PRF versus Geistlich Bio-Gide^®^) [23]. In both studies, included subjects were systemically healthy adults with maxillary atrophy (defined as <5 mm residual bone crest height measured in OPG/orthopantomogram). Smoking status was not assessed. The addition of PRF to Bio-Oss^®^ bone substitute revealed neither advantages nor disadvantages over Bio-Oss^®^-alone controls [22]. After six months, clinical and radiographic examinations revealed both groups exhibiting similar amounts and density of mineralized tissues, with no signs of material resorption. Histological evaluations also showed non-significant differences regarding: (a) newly generated bone percentage; (b) residual Bio-Oss^®^ percentage; (c) bone-to-bone-substitute contact; and (d) post-treatment inflammatory reactions [22]. Regarding coverage of the lateral osteotomy sinus window, PRF use resulted in a comparable amount of residual bone substitute and vital bone formation (%) when faced against Bio-Gide^®^ controls (PRF: 17.0% and 15.9%, Bio-Gide^®^: 17.2% and 17.3%, differences are not statistically significant). Overall, despite a slightly superior to no coverage at all (12.1%), it can be stated that results were similar to those reported using other conventional membranes (collagen: 17.6%; e-PTFE (expanded polytetrafluoroethylene): 16.9%) [23]. Within the presented limitations in both RCTs, evidence suggests that PRFs are safe, simple to use and handle, and a cost-effective alternative to traditional bone grafts and absorbable membranes for low-income patients pursuing maxillary sinus augmentation procedures.

### 3.5. PRFs in Alveolar Ridge Preservation

Post-extraction changes in alveolar bone compromise prosthodontic rehabilitation with fixed, removable and/or implant-supported prosthesis. Alveolar ridge preservation (ARP) is a technique that involves the application of grafting and barrier materials in order to significantly reduce post-extraction bone loss [31]. It has been demonstrated that PRFs are able to accelerate/enhance bone repair [32,33], promote fibroblast proliferation [3,33], and increase vascularity [24], thereby potentially favoring the post-extraction healing process and the ARP approach. Yet, a single RCT evaluating the use of PRF in ARP was identified, according to the inclusion criteria set herein [34]. This sole study compared the application of PRF versus natural blood clots in post-extraction sockets of third molars. Patients were systemically healthy and non-smoking adults requiring bi-lateral mandibular third molar removal. The use of PRF significantly improved post-extraction soft tissue healing after seven days [34]. Early and significantly higher radiographic bone formation/maturation was noticed in the PRF-treated sites versus controls at eight weeks. By 12 weeks, intergroup differences were non-significant. Radiographic bone density (measured by gray scale value) at 12 weeks increased in the biomaterial group compared with the controls; nonetheless, the differences were not significant [34]. Similar to other studies, PRF reduced early post-surgical pain (VAS scale: Visual Analogue Scale) on day 1; however, intergroup differences were not significant by day 7 [34].

### 3.6. Clinical Expertise with L-PRF in Periodontally Accelerated Osteogenic Orthodontics

In the pilot phase of our own prospective observational study [33], including 11 patients into the cohort (with informed consent) receiving a Wilcko’s modified PAOO (periodontally accelerated osteogenic orthodontics—*method that combines orthodontic efforts with corticotomy and grafting of alveolar bone plates allowing faster tooth movement, which is considered a rather new surgical practice*) approach alongside L-PRF (added as covering membrane and into the graft), accelerated wound healing without evident signs of infection or adverse reactions (Figure 4D). For 10 days, post-operative pain, inflammation, and infection were recorded, while the follow-up of the overall orthodontic treatment and post-treatment stability lasted two years. In our data analysis, post-surgical pain and immediate inflammation after surgery were found to be either “mild”, with 45.5% for the first one and 89.9% for the second, or “moderate”, with 54.5% and 9.1%, respectively; and the majority of patients suffered either “mild” or no inflammation (72.7% and 9.1%, respectively) on day 4, where settlement was marked to begin. Interestingly, by day 8, a complete resolution was achieved in all patients; the average orthodontic treatment time was calculated at 9.3 months, and all of the cases were stable throughout. Thus, we concluded that combining L-PRF with traditional bone grafts (L-PRF plug or block) possibly stimulates the healing of the wound and diminishes pain after surgery, inflammation, and infection with no interference in the movement of the tooth and/or stability after orthodontic treatment over a two-year observational extent of time; thereby, the demand for analgesics and anti-inflammatory medications is alleviated [33]. Our recommended preparation protocol for reproducible and clinically usable and malleable L-PRF clots and membranes is defined in Table 2.

## 4. Conclusions and Closing Remarks

Tissue regeneration and anatomical reconstruction and repair in defects of the oromaxillofacial complex have been consistently a critical and controversial concern. Both quality and quantity of the regenerated tissues are important to consider, aesthetically and functionally. Practically, the surgeon is faced with an ample collection of regenerative techniques and materials to choose from. *How can one select the “ideal” or “best fit” strategy and procedure to meet the most favorable clinical outcome? Evidence-based studies? Level of scientific evidence?* This review, to the best of our knowledge, is the first concerning randomized controlled clinical trials on PRFs’ use and application in specific oral and maxillofacial surgery procedures, bridging the gap between the clinical and the materials expert. While the available literature is found to be highly limited, PRFs *can still be* indicated as an innovative *tool* for contemporary oromaxillofacial tissue regeneration and bioengineering. Indeed, the existing evidence suggests that PRFs improve early wound healing and promote post-surgical bone formation/maturation. However, it is noteworthy that a clearer consensus seems to be present regarding its significant beneficial impact on post-surgical pain and discomfort control, regardless of the type of procedure. Unlike its predecessors, new PRF preparations tend to function more as biologically active biomaterials and scaffolds for autologous cell, growth factors, and cytokines delivery. Thus, PRFs should be considered a “living tissue” preparation for natural guided tissue regeneration, and not simply a “growth factor-rich” surgical adjuvant. Yet, it is safe to say that this remains an unexplored territory in dental biomaterial (Dental BioEngineering) research in general. 

Currently, our group is investigating the potential of (a) incorporating mesenchymal stem cells derived from orodental routes and (b) core-shell nanoparticles loaded with growth factor(s) and incorporated or embedded within the PRFs as “*super*” or “*smart*” bio-scaffolds to further promote soft tissue healing, bone generation, treatment time, post-surgical stability, and outcome predictability, in advanced oromaxillofacial surgical procedures such as periodontally accelerated osteogenic orthodontics (PAOO) and mental genioplasty [35]. Further, in the context of invasive surgical procedures such as third molar extraction and cysts resections, the ultimate objective of our research focuses on the potential of PRFs in decreasing the need for prescription drugs. Finally, we are vigorously working on characterizing the rheological properties and biological variations of PRFs, alongside partnering up with nurses, physicians, and dentists to standardize the preparation protocol for use in other therapeutic indications, including orthopedics, in an effort to address and resolve the underlying challenges and limitations outlined earlier herein that are slackening their clinical translation. 

## Figures and Tables

**Table 1:** Summary of clinical literature (randomized clinical trials, or RCTs) on L-PRF use in oral and maxillofacial Surgery. IBD: intra-bony defects.

Application	No. Patients/Defects	Groups	Follow-up (Months)	Main Finding(s)	Reference
**IBD**	32/32	(1) PRF + open flap surgery (2) Open flap surgery	9	All sites healed uneventfully. Probing depth (PD) reduction, average clinical attachment (CAL) gain, defect fill, percentage defect fill and post-treatment gingival margin stability were significantly greater in the PRF-treated group. (*P* < 0.05).	[9]
**IBD**	15/30	(1) PRF + open flap surgery (2) Open flap surgery	12	All sites healed uneventfully. PD reduction, CAL gain, radiographic IBD depth reduction, and post-treatment gingival margin stability were significantly higher in the PRF group. Statistically significant higher patient acceptance and healing index in PRF vs. control.	[10]
**IBD**	35/56	(1) PRF + open flap surgery (2) Open flap surgery	9	All sites healed uneventfully. PD reduction, CAL gain, radiographic IBD defect fill were significantly higher in the PRF group. Gingival Margin Stability (GMS) was higher in the PRF group.	[11]
**IBD**	17/34	(1) PRF + Bio-Oss^®^ (2) PRF	6	All sites healed uneventfully. Both groups showed significant PD reduction, CAL gain, and IBD fill. Intergroup differences were also significant and favored the PRF/Bio-Oss group.	[12]
**IBD**	10/20	(1) PRF + DFDBA (2) DFDBA (demineralized-freeze dried bone allograft)	6	Both groups experienced significant PD reduction, CAL gain, IBD fill, and IBD resolution. Intergroup differences were statistically significant only for PD reduction and CAL gain, favoring the PRF/DFDBA group.	[13]
**IBD**	21/21	(1) PRF + inorganic bovine bone mineral (2) Anorganic bovine bone mineral	6	All of the sites healed uneventfully with no clinically detectable or subjectively reported side effects. Both treatment groups showed significant improvements compared to baseline in terms of vertical bone gain, defect fill, and defect angle at six months after treatment (*P* < 0.05). Addition of PRF to inorganic bovine bone mineral (ABBM: Anorganic bovine bone mineral) may lead to the enhancement of clinical attachment level gain.	[14]
**IBD**	16/32	(1) Resorbable collagen membrane + PRF (2) Guidance tissue regeneration	9	Test group showed a statistically significant improvement for probing depth (*P* = 0.002), clinical attachment level (*P* = 0.001), and radiographic defect depth (*P* < 0.001) after nine months as compared with the control sites. The adjunctive use of PRF in combination with barrier membrane is more effective in the treatment of intrabony defects in chronic periodontitis as compared with barrier membrane alone.	[15]
**PFD (Periodontal Furcation Defect**)	18/38	(1) PRF + open flap surgery (2) Open flap surgery	9	All sites healed uneventfully. No significant visual differences between groups were noticed. Complete clinical closure was achieved in 66.7% of the defects in the PRF group. Within residual furcation defects, 5/6 were reduced from grade II to grade I, and one defect remained grade II. Significantly greater PD reduction, CAL gain, and defect fill was noticed in the PRF-treated group vs. control.	[16]
**Gingival Recession**	15/30	(1) PRF + coronally-advanced flaps (CAF) (2) CAF	6	Both groups experienced statistically significant recession depth (RD) reduction, CAL gain, and keratinized tissue width (KTW) increase at all time intervals (*P* < 0.05). Intergroup differences were statistically significant and favored the PRF group. Mean percentage of root coverage for the test and control groups were 100% and 68.44%, respectively. Differences between groups were statistically significant and favored the PRF group.	[17]
**Gingival Recession**	20/67	(1) PRF + CAF (2) CAF	6	With the exception of CAL gain and gingival tissue thickness (GTH) increase, the addition of PRF to CAF failed to produce significant additional clinical benefits (vs. CAF-alone). Percentage root coverage, full root coverage, GMS, and recession width (RW) reduction were significantly higher in the CAF controls than the PRF-treated sites after six months.	[18]
**Gingival Recession**	20/40	(1) PRF + CAF (2) EMD + CAF	12	Both groups experienced statistically significant RD reduction, PD reduction, and KTW increase. Intergroup differences were significant only for KTW increase and favored the enamel matrix derivate (EMD) group. Mean root coverage was 70.5 ± 11.76% in the EMD group, and 72.1 ± 9.55% in the PRF group. Complete root coverage was achieved in 60% of the EMD sites and 65% of the PRF sited. No intergroup comparison was carried out. The healing index of the PRF group after the first week was significantly superior to that of EMD. Non-significant differences between groups were found after two weeks post-surgery. Three patients of the EMD group and one of the PRF group experienced severe pain. All of the patients in the EMD group reported greater discomfort. Analysis of the first five days post-surgery revealed statistically significant differences between both groups favoring PRF (less pain).	[19]
**Gingival Recession**	22/44	(1) PRF + CAF (2) SCTG(subepithelial connective tissue graft) + CAF	6	Both groups experienced a statistically significant decrease in RD (Gingival recession depth), RW (Gingival recession width), and RA (Gingival Recession Area), plus an increase in CAL (Clinical Attachment Level) gain, KTW (Keratinized Tissue Width), and GT (Gingival Thickness). Intergroup differences were non-significant. Higher yet non-significant gingival margin stability was reported for the PRF group. Percentage of root coverage and complete root coverage were 92.7% and 72.7% in the test group and 94.2% and 77.3% in the control group. No statistical significant differences between both groups were found (*P* > 0.05). All of the sites healed uneventfully; however, the control group reported complications (i.e., pain) related to the palate donor site.	[20]
**Gingival Recession**	15/30	(1) PRF + CAF (2) Connective tissue grafts (CTG) + CAF	6	Both groups experienced a significant CAL gain, RD reduction, and GMS. Intergroup differences were non-significant. Both groups experienced a statistically significant increase in KTW. Intergroup differences were statistically significant and favored the CTG group. Mean root coverage was 88.68 ± 10.65% for the PRF group and 91.96 ± 15.46% for the control group. Complete root coverage was achieved in 75.85% of cases in the PRF group and 79.56% of cases in the control group. Intergroup differences were non-significant. Healing index values of the PRF group during the first two weeks were statistically superior to those of the CTG control. One patient from the PRF group and seven from the CTG group experienced severe pain. Also, all of the patients in the control group reported some discomfort. Pain intensity was statistically superior in CTG during the first week.	[21]
**Maxillary Sinus Augmentation (Graft)**	10/11	(1) PRF + Bio-Oss^®^ (2) Bio-Oss^®^	6	Healing was uneventful for all patients. Both groups exhibited an adequate amount and density of radiographic mineralized tissue plus a similar composition, distribution, and inflammation of histological structures. Intergroup differences were non-significant. The percentage of newly formed bone was about 1.4 times greater in the PRF group (18.35 ± 5.62% vs. 12.95 ± 5.33% of control). The percentage of residual bone substitute material was about 1.5 times greater in the control group (28.54 ± 12.01% vs. 19.16 ± 6.89% of LPRF). The bone-to-bone substitute contact was 21.45 ± 14.57% and 18.75 ± 5.39% in the PRF and the control group, respectively. Intergroup differences were non-significant.	[22]
**Maxillary Sinus Augmentation (Membrane)**	6/12	(1) PRF (2) Bio-Gide^®^	5	Wound healing was uneventful for all patients. No soft tissue in-growths were observed in both groups. Surfaces seemed homogenous with visible bone-substitute material embedded into newly-formed bone. The average amount of vital bone and bone substitute were 17.0% and 15.9% in the PRF group. Control group had 17.2% and 17.3%. No intergroup comparisons were carried out.	[23]
**Alveolar Socket Preservation**	20/40	(1) PRF (2) Empty (blood clot)	3	Soft tissue healing was significantly better in the PRF group vs. the controls (Laundry, Turnbell and Howley Soft Tissue Healing Index). Early bone formation/maturation was noticed for the experimental sites vs. controls. Differences were significant only at eight weeks post-extraction and favored the PRF group. Higher bone density was noticed in the PRF group vs. controls. Intergroup differences were non-significant. Mean post-surgical pain (measured by Visual Analogue Scale (VAS) score) was reduced in the PRF group vs. non PRF controls at day 1. By day 7, no intergroup differences were noticed.	[24]

**Table 2:** Author’s recommendations for L-PRF preparation.

Recommended Preparation Protocol for Clinically-fit L-PRF (Clots, Plugs, Blocks and Membranes)	
Collect 5–9 mL whole venous blood sample into 2–3 sterile 6 mL glass-coated plastic vacutainer tubes (without anti-coagulant -clot formation).	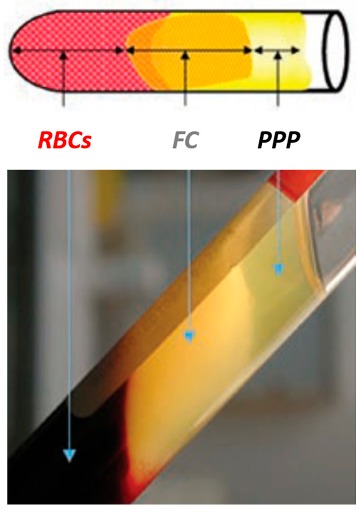
Centrifuge immediately for 10–12 min at 2700–3000 rpm (revolutions per minute) using any high-quality table-top centrifuge.	
Fibrinogen → Fibrin. Typically, 3 distinct compartments should be evident in each tube. UPPER Portion: straw-colored acellular plasma (PPP); MIDDLE Portion: yellowish fibrin clot (FC); and LOWER Fraction: red-colored corpuscles of red blood cells (RBCs).	
Quickly remove the upper layer to reveal and collect the middle portion; around 2 mm below the lower dividing line. Timing is critical to obtain bioactive L-PRFs charged with serum and platelets. This clot can then be used directly, either (a) as filling material; (b) mixed with bone grafting materials(s) – plugs and blocks; and/or (c) compressed (using the surgical box to prevent damage and to collect fibrin surgical glue in reservoir) into a strong and resilient clinically-usable membrane. For injectable PRF or iPRF (a more liquid or flowable formulation), centrigue tube again for 3–4 more minutes and collect top 1 mL layer using a syringe suitable for immediate injection into the intended application site. Mixing with other biomaterials is feasible as well. Note: slower centrifugation (less than 1500 rpm)for less time period (around 6–8 min) will result in a preparation with higher white blood cell count, commonly termed Advanced PRF or A-PRF suitable for defects requiring more vascularization (5 min needed to induce fibrin clot formation).	
